# Effect of a mattress on lumbar spine alignment in supine position in healthy subjects: an MRI study

**DOI:** 10.1186/s41747-023-00361-w

**Published:** 2023-09-04

**Authors:** Jacopo Antonino Vitale, Stefano Borghi, Tito Bassani, Carmelo Messina, Luca Maria Sconfienza, Fabio Galbusera

**Affiliations:** 1 Spine Center, Schulthess Klinik, Zurich, Switzerland; 2 Department of Biomedical Sciences for Health, University of Milan, Milan, Italy; 3 IRCCS Istituto Ortopedico Galeazzi, Milan, Italy

**Keywords:** Lordosis, Magnetic resonance imaging, Sacrum, Sleep, Spine

## Abstract

**Background:**

Humans should sleep for about a third of their lifetime and the choice of the mattress is very important from a quality-of-life perspective. Therefore, the primary aim of this study was to assess the changes of lumbar angles, evaluated in a supine position using magnetic resonance imaging (MRI), on a mattress *versus* a rigid surface.

**Methods:**

Twenty healthy subjects (10 females, 10 males), aged 32.3 ± 6.5 (mean ± standard deviation), with body mass index 22.4 ± 2.9, completed three evaluations: (i) spine MRI in supine position on a mattress (MAT); (ii) spine MRI in supine position on rigid surface (CON); and (iii) biplanar radiographic imaging in standing position. The following indexes were calculated for both MAT and CON: lumbar lordosis angles L1–L5, L1–S1, L5–S1, and the sacral slope (SS). Further, pelvic incidence (PI) was calculated from the biplanar radiographic images.

**Results:**

Main findings were (i) L1–L5 and SS were greater in MAT than CON (L1:L5: +2.9°; SS: +2.0°); (ii) L5–S1 was lower in MAT than CON (−1.6°); (iii) L1–S1 was greater in MAT than CON only for male subjects (+2.0°); (iv) significant and positive correlations between PI and L1–L5, L1–S1 and SS were observed in both CON and MAT.

**Conclusions:**

The use of a mattress determined small but statistically significant changes in lumbar angles.

**Relevance statement:**

The use of a mattress determines small but statistically significant changes in radiological angles describing the sagittal alignment of the lumbar spine when lying in the supine position.

**Key points:**

• Lordosis angle L1–L5 was greater in MAT than in CON condition (+2.9°).

• Sacral slope was greater in MAT than in CON condition (+2.0°).

• Lordosis angle L5–S1 was lower in MAT than in CON condition (−1.6°).

**Graphical Abstract:**

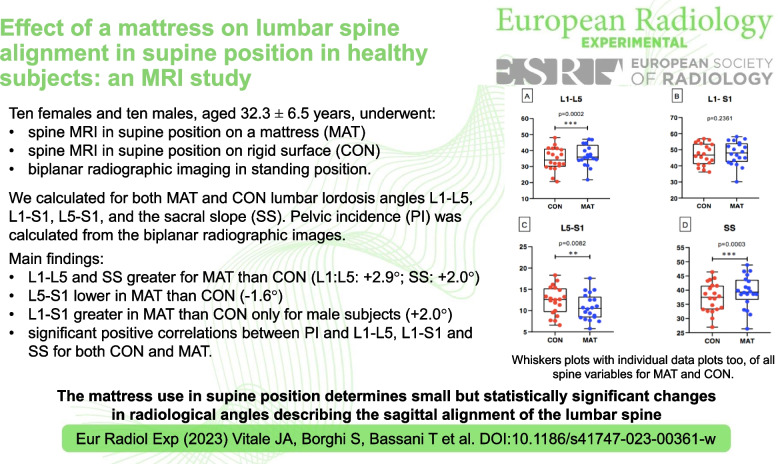

## Background

Sleep is an essential biological process with a myriad of psychophysiological functions [1]. Humans should sleep for about a third of their lifetime (*i.e.*, 7−9 h a day); however, 30−50% of the general population reports sleep problems such as insomnia symptoms, short sleep duration, or low sleep quality [2, 3].

The National Sleep Foundation, USA, highlighted that 93% of people recognize a comfortable mattress as an important instrument being able to get quality sleep [4]. Thus, the choice of the mattress is very important from a quality-of-life perspective [5]. In a survey conducted by orthopedic surgeons and addressed to patients diagnosed with low back pain following sleep, it was observed that 95% of participants considered the mattress important in the management of low back pain and 75% recommended mattresses of moderately hard or medium rigidity to relieve back pain [6, 7]. Therefore, sleeping supports are considered important environmental components influencing physical comfort during sleep and thus affecting health [8].

The mechanical characteristics of the mattress are crucial for sleep quality and body comfort. If the mattress is too soft, the mechanical support to musculotendinous structures may be lower leading to higher tension to posterior soft tissue elements, while the intervertebral discs will be under tension at the anterior side. Conversely, if the mattress is too firm, the lumbar section of the spine will not smoothen immediately when lying down [9]. Since spinal alignment or curvature and contact pressure are the predominant variables of interest, it is important to know their value/desirable range to reach the optimization of design and realize high-quality mattresses [8].

Different methods have been described in literature to evaluate bed comfort, such as spine shape reconstruction [10, 11], electromyography [12], pressure mapping [13], and subjective evaluations [14]. Magnetic resonance imaging (MRI), being the technique of choice in the multicompartmental evaluation of the spine, including bone, discs, nerves, and soft tissues [15], is the ideal method to assess the behavior of the spine in lying patients. In a study of Mauch et al. [15], the subjects were examined using MRI while lying recumbently (supine) and while standing in a weight-bearing position. The analysis of the two positions showed a high significant increase in lumbar lordosis in the weight-bearing position (approximately +6.3°). On the contrary, Hirasawa et al. [16] studied the lordosis L1–S1 angle in supine and standing positions with the same method, showing no significant differences between conditions. In any case, the assessment of spinal alignment in the supine position on the bed remains challenging because of the lack of back exposure and the fact that instrument placement may interfere with body support.

To the best of our knowledge, no previous studies examined the changes in spine angles in healthy adults while lying down on surfaces with different rigidities in a supine position. Therefore, the primary aim of this study was to assess the changes of the L1–L5, L1–S1, L5–S1, and sacral slope (SS) angles, evaluated by MRI, between two conditions: (1) in a supine position on a mattress; (2) in a supine position on hard surface. The second aim was to analyze the correlation between the spine angles evaluated in a standing position, using the EOS system, and L1–L5, L1–S1, L5–S1, and SS angles in both supine conditions (mattress and control). We hypothesized to detect significant changes in spine angles between mattress and control conditions.

## Methods

### Study design

This observational, cross-sectional, pilot study was approved by the Ethics Committee of San Raffaele Hospital (Ref. 158/INT/2020). All procedures were performed in compliance with current national and international laws and regulations governing the use of human subjects (Declaration of Helsinki). The study protocol was registered at clinicaltrials.gov (Ref. No. NCT04638374). All subjects received clear explanation of purpose, methods, potential risks, and benefits of the study, and before the beginning of the experimental procedures, written informed consent was obtained from all participants. The study was conducted at the IRCCS Istituto Ortopedico Galeazzi (Milan, Italy), in accordance with the STROBE guidelines [17] for cross-sectional studies, between February 2021 and May 2021.

### Subjects and biometric data

Subjects were invited to participate in the study at the Radiology Service of IRCCS Istituto Ortopedico Galeazzi, Milan, Italy. Exclusion criteria were recent fracture; surgery within the past 12 months; history of low back pain in the previous 12 months; spinal disorder including degenerative disease and deformities (*e.g.*, scoliosis); contraindications to MRI (*e.g.*, pacemaker, claustrophobia). Therefore, healthy subjects aged between 18 and 45 years old who met the inclusion criteria were included in the study. All subjects completed the following clinical evaluations to assess the spine angles: (i) spine MRI in supine position on a mattress (MAT); (ii) spine MRI in supine position on hard surface, as control condition (CON); (iii) EOS imaging (EOS system, see below for details).

The order of execution of imaging MAT and CON conditions was randomized. Before MRI, height and weight data were obtained using a mobile stadiometer (Seca 217; Vogel & Halke, Hamburg, Germany). Height was rounded to the nearest 1 cm and body mass to the nearest 0.5 kg. Body mass index (BMI) was calculated using the standard formula (weight in kilograms divided by height in meters squared).

### Mattress material and size

**Fig. 1:**
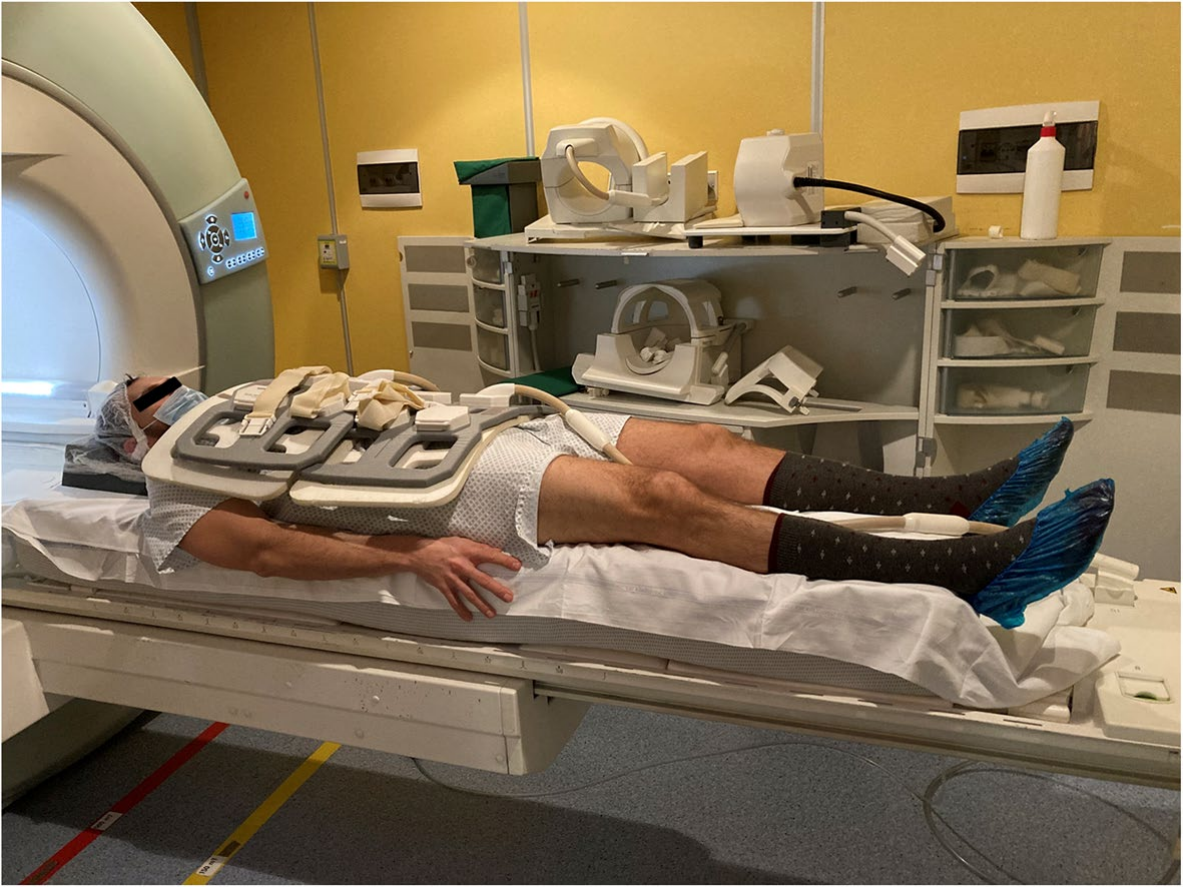
A study subject laying down on the mattress before MRI acquisition

The mattress used in this study was a medium firm mattress composed of a single layer of polyurethane (Dorelan, B&T SpA, Forlì, Italy). The mattress size was adapted for the 1.5-T MRI scanner (Espree, Siemens Healthineers AG, Erlangen, Germany); in detail: 50 cm width and 190 cm length. Total mattress thickness was 22 cm. The rigid surface utilized in this study was the standard MRI scanning bed. Figure 1 shows a study subject laying down on the mattress before MRI acquisition.

### MRI

Standard T2-weighted sagittal images of the lumbar spine were performed with turbo spin-echo sequences for the assessment of the radiological angles (repetition time/echo time = 4,100/102 ms; slice thickness = 4 mm; number of excitations = 2). The following indexes were calculated for each subject on the midsagittal slice in both MAT and CON: (i) lumbar lordosis angle (L1–L5) [18]; (ii) L1–S1 angle (L1–S1) [16]; and (iii) sacral slope (SS) [19]. Further, the difference between L1-S1 and L1–L5 was calculated to obtain the L5–S1 angle. The angles were assessed by using the manual measurement tools provided by the Picture Archiving and Communication System (IDS7, SectraAB, Linköping, Sweden).

### EOS

Digitized images of the thoracolumbar spine and pelvis were performed with the EOS Imaging System (EOS Imaging, Paris, France), which simultaneously acquires images in coronal and sagittal planes, with subjects in standing position. No further calibration procedures were required. The following indexes were calculated for each subject, again using the IDS7 system measurement tools: (i) L1-L5 angle; (ii) L1**–**S1 angle; (iii) SS, *i.e.*, the angle between the horizontal line and the superior endplate of the sacrum; and (iv) pelvic incidence (PI), *i.e.*, the angle between the line perpendicular to the sacral plate and the line connecting the midpoint of the sacral plate to the midpoint of the femoral heads.

### Statistical analysis

Statistical analysis was performed using GraphPad Prism version 6.00 (GraphPad Software, San Diego, CA, USA).

#### Baseline characteristics

The normality of the distribution of the anthropometric and demographic variables (weight, height, BMI and age), both for the entire study sample and for male and female subjects separately, was checked using graphical methods and the Shapiro–Wilk test. Since all variables were normally distributed, the baseline differences between genders were checked with unpaired Student’s *t* test.

#### Intra- and inter-rater reliability

Four different investigators (J.V., S.B., L.M.S., and F.B.) manually performed all measurements on MRI and EOS images for all subjects and the analysis was repeated two times, a month away, by only one investigator (S.B.). Intra- and inter-rater reliability was tested for each studied outcome in MAT and CON. For the inter-rater reliability, intraclass correlation coefficient (ICC) estimates and their 95% confidence intervals (CIs) were calculated based on a single rating, consistency, 2-way mixed-effects model. Further, for the intra-rater reliability, ICC estimates, and their 95% CIs were calculated based on a single rating, absolute agreement, 2-way mixed-effects model. As previously described [20], values less than 0.5 were considered indicative of poor reliability, values between 0.5 and 0.75 of moderate reliability, values between 0.75 and 0.9 of good reliability, and values greater than 0.90 of excellent reliability. ICCs were calculated with MATLAB (MathWorks Inc., Natick, MA, USA).

#### Mattress versus rigid surface

The normality of the distribution of each MRI and EOS measurements, both for the entire study sample and for male and female subjects separately, was checked using graphical methods and the Shapiro–Wilk test. All variables were normally distributed with the exception for L1**–**L5 and SS, evaluated by the EOS system, for females. Differences between MAT and CON were tested through paired Student’s *t* test; further, delta values (MAT minus CON) were calculated for males and females and were compared using unpaired Student’s *t* tests or with the Mann–Whitney rank test for non-normally distributed variables. Significance was set at *p* < 0.05. Effect sizes (ES) were used to determine the magnitude of the effect for all significant outcomes of pairwise comparison using Cohen’s *d* and considered to be either trivial (< 0.20), small (0.21–0.60), moderate (0.61–1.20), large (1.21–2.00), or very large (2.00) [21].

#### Correlation analysis

The existence of a correlation between PI, as evaluated by the EOS system, and L1**–**L5, L1**–**S1, L5**–**S1, and SS in MAT or CON (and delta values too) was tested by the means of the Pearson correlation coefficient. Correlations were considered significant when *r* > 0.25 and *p* < 0.05.

## Results

### Participants’ characteristics at baseline

Twenty-one subjects met the inclusion criteria and were included in the study (10 females, 11 males). One male subject was dropped a posteriori because of the presence of spondylolisthesis at L3**–**L4 level as diagnosed by an expert radiologist (L.M.S.). Data on age, height, body mass, BMI, and spine angles evaluated in a standing position by the EOS are presented in Table 1.

**Table 1:** Subjects’ characteristics at baseline

	Total (*n* = 20)	Females (*n* = 10)	Males (*n* = 10)	*p* values
Age (years)	32.3 ± 6.5	31.8 ± 6.7	32.7 ± 6.6	0.767
Height (cm)	172.2 ± 8.0	167.6 ± 6.0	176.8 ± 7.0	0.003
Body mass (kg)	66.8 ± 11.4	58.8 ± 5.9	74.7 ± 9.9	< 0.001
BMI (kg/m^2^)	22.4 ± 2.9	20.9 ± 1.4	23.9 ± 3.4	0.021
L1–L5 (degrees)	45.2 ± 8.6	46.0 ± 9.2	44.4 ± 8.4	0.677
L1–S1 (degrees)	56.4 ± 8.5	57.6 ± 7.4	55.3 ± 9.7	0.248
SS (degrees)	36.9 ± 8.6	38.3 ± 10.6	35.6 ± 6.4	0.166
PI (degrees)	48.4 ± 9.6	47.0 ± 11.8	49.8 ± 7.3	0.525

*BMI* Body mass index, *PI* Pelvic incidence, *SS* Sacral slope. Spine angles were evaluated with subjects in a standing position by the EOS system

### Reliability and ICCs

Table 2 shows the inter-rater and intra-rater ICCs and their 95% CI for each variable in MAT or CON. Intra-rater ICCs were classified as excellent (100%) and inter-rater ICCs were classified as good (70%) or excellent (30%).

**Table 2:** Inter-rater and intra-rater intraclass correlation coefficients (ICCs) and their 95% confidence intervals (CIs)

	Inter-rater ICC (95% CI)	Intra-rater ICC (95% CI)
L1–L5 MAT	0.822 (0.624–0.924), good	0.963 (0.908–0.985), excellent
L1–L5 CON	0.882 (0.747–0.950), good	0.967 (0.920–0.987), excellent
L1–S1 MAT	0.865 (0.759–0.937), good	0.980 (0.950–0.992), excellent
L1–S1 CON	0.903 (0.812–0.957), excellent	0.927 (0.818–0.971), excellent
SS MAT	0.751 (0.585–0.877), good	0.963 (0.908–0.985), excellent
SS CON	0.905 (0.816–0.958), excellent	0.952 (0.872–0.982), excellent
L1–L5 EOS	0.859 (0.666–0.943), good	0.980 (0.950–0.992), excellent
L1–S1 EOS	0.907 (0.810–0.960), excellent	0.968 (0.920–0.987), excellent
SS EOS	0.892 (0.757–0.955), good	0.958 (0.833–0.986), excellent
PI EOS	0.891 (0.767–0.953), good	0.959 (0.883–0.985), excellent

*CON* Control, *EOS* EOS System, *MAT* Mattress, *PI* Pelvic incidence, *SS* Sacral slope

**Fig. 2:**
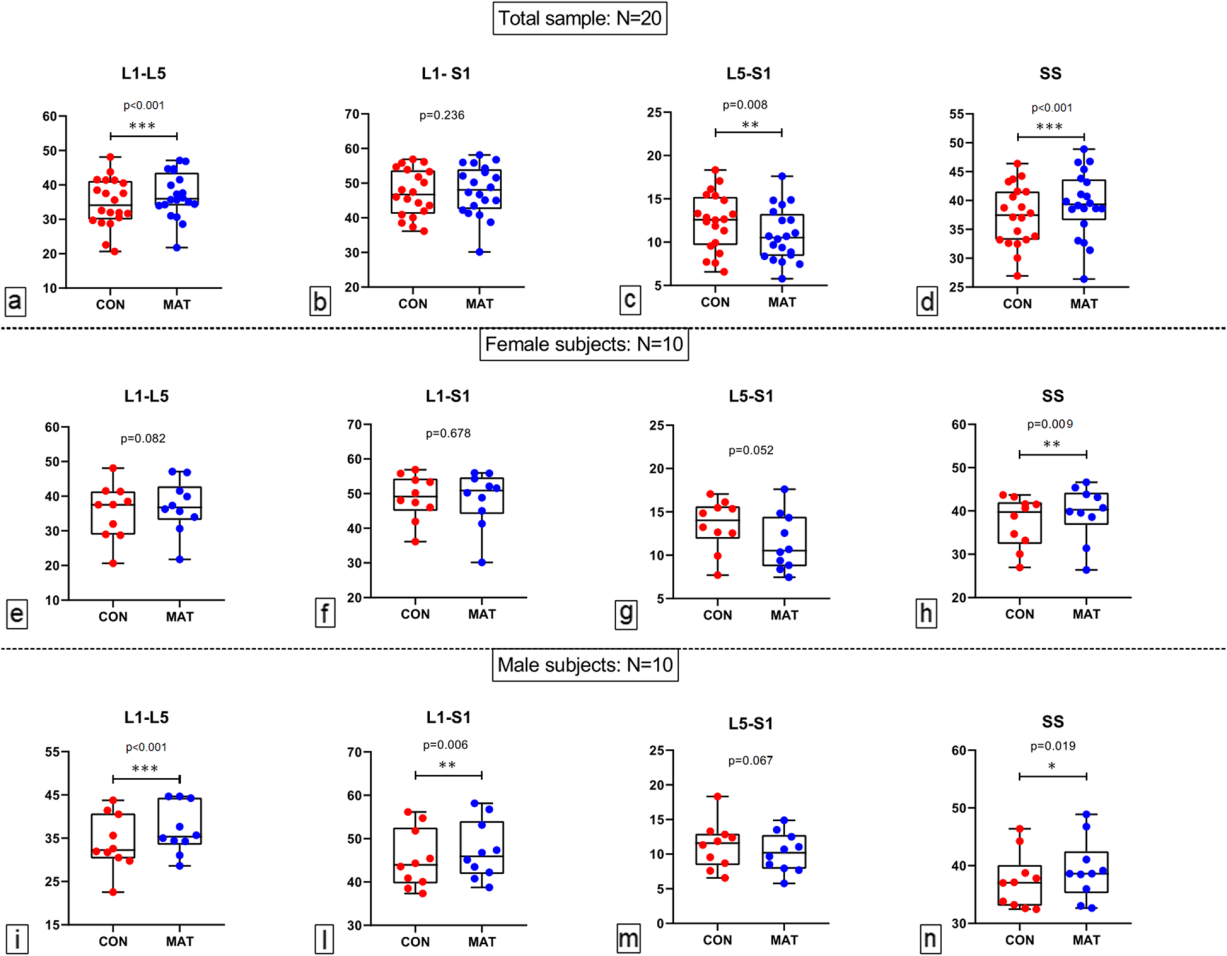
Whiskers plots, with individual data plots too, of all spine variables in MAT and CON. *CON* Control, *MAT* Mattress

Table 3 shows the comparison between MAT and CON and multipanel Fig. 2 displays the whiskers plots, with individual data plots too, of all spine variables in MAT and CON. L1**–**L5 was greater in MAT than CON (+2.9°) with the only exception for female subjects whereas L1-S1 angle was +2.0° greater in MAT than CON only for males (*p* = 0.006; ES 0.30, small). SS was always significantly greater in MAT than CON (+2.0°) and, conversely, L5**–**S1 was lower in MAT than CON only for the entire sample (−1.60°, *p* = 0.008; ES 0.50, small). Further, no significant differences were observed in the comparisons of delta values between male and female subjects (L1**–**L5, *p* = 0.179; L1**–**S1, *p* = 0.052; L5**–**S1: *p* = 0.630; SS: *p* = 0.895).

**Table 3:** Comparison between MAT and CON for all MRI-based calculated spine angles

	CON	MAT	Difference	*p* value	ES
Total (*n* = 20)					
L1–L5	34.7 ± 7.1	37.6 ± 6.5	+2.9	< 0.001	0.34, small
L1–S1	47.1 ± 6.7	47.9 ± 7.2	+0.8	0.236	NA
L5–S1	12.4 ± 3.3	10.8 ± 3.1	−1.6	0.008	0.50, small
SS	37.4 ± 5.2	39.4 ± 5.6	+2.0	< 0.001	0.37, small
Females (*n* = 10)					
L1–L5	35.5 ± 7.9	37.1 ± 7.5	+1.6	0.082	NA
L1–S1	49.0 ± 6.5	48.5 ± 8.0	−0.5	0.678	NA
L5–S1	13.5 ± 2.9	11.4 ± 3.3	−2.1	0.052	NA
SS	37.4 ± 5.9	39.5 ± 6.3	+2.1	0.009	0.34, small
Males (*n* = 10)					
L1–L5	34.0 ± 6.4	37.0 ± 5.7	+3.0	< 0.001	0.50, small
L1–S1	45.3 ± 6.7	47.3 ± 6.7	+2.0	0.006	0.30, small
L5–S1	11.3 ± 3.4	10.2 ± 2.9	−1.1	0.067	NA
SS	37.3 ± 4.8	39.3 ± 5.2	+2.0	0.019	0.40, small

Data are degrees reported as mean ± standard deviation. *CON* control, *ES* effect size (Cohen d), *MAT* Mattress, *MRI* Magnetic resoance imaging, *NA* Not applicable, *SS* sacral slope

**Fig. 3:**
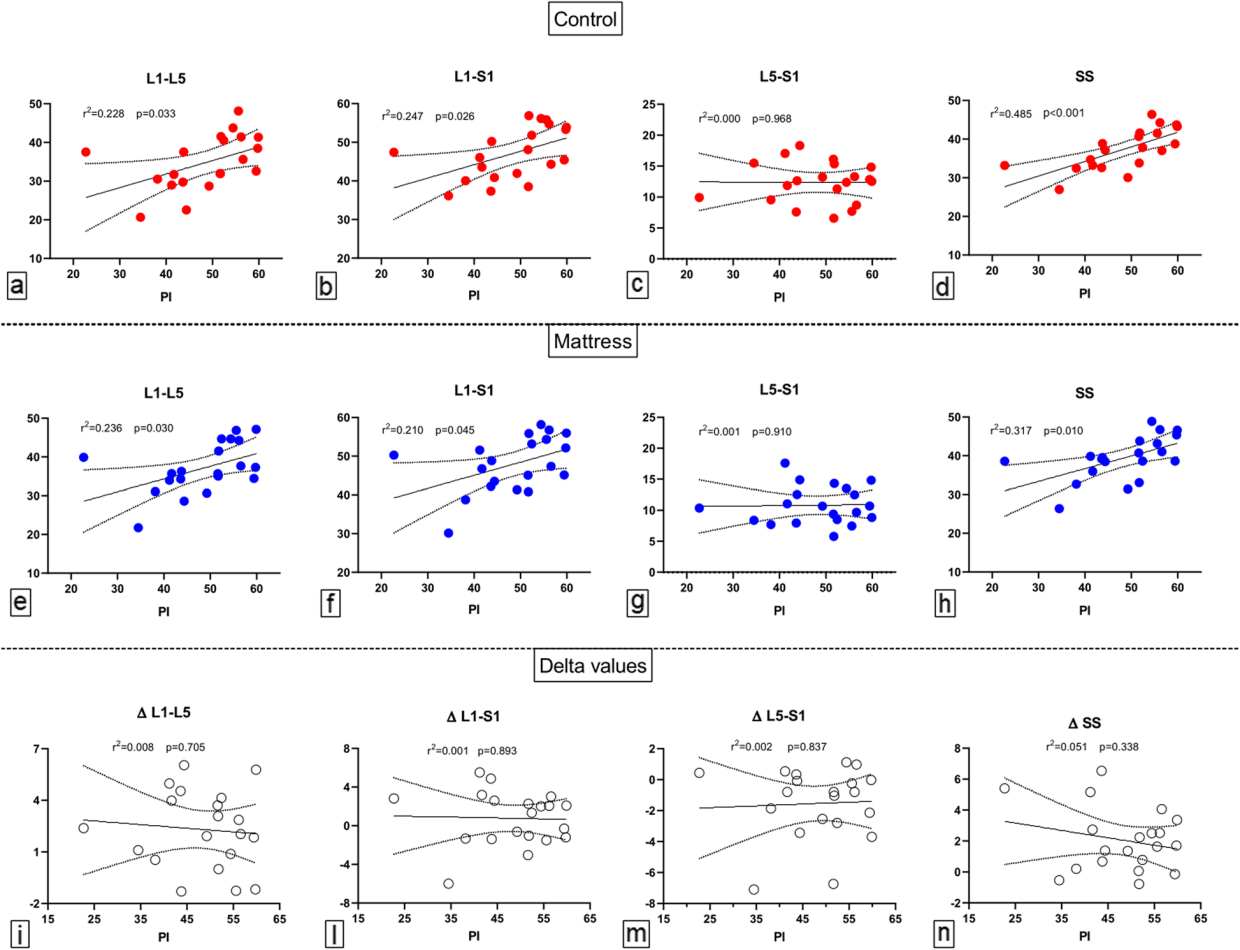
The correlation between PI and L1–L5, L1–S1, L5–S1 angles and SS for CON and MAT, and associated delta values. *PI* Pelvic incidence, *SS* Sacral slope

Multipanel Fig. 3 graphically shows the correlation between PI and L1**–**L5, L1**–**S1, L5**–**S1, and SS in CON, MAT, and associated delta values. Significant and positive correlations between PI and L1**–**L5, L1**–**S1, and SS were observed both in CON (L1**–**L5, *r*^2^ = 0.228 and *p* = 0.033; L1**–**S1, *r*^2^ = 0.247 and *p* = 0.026; SS, *r*^2^ = 0.485 and *p* < 0.001) and MAT (L1**–**L5, *r*^2^ = 0.236 and *p* = 0.030; L1**–**S1, *r*^2^ = 0.210 and *p* = 0.045; SS, *r*^2^ = 0.317 and *p* = 0.010) whereas no significant correlations between PI and L5**–**S1, both in MAT and CON, and between PI and delta values were detected.

## Discussion

The lack of sleep negatively impact an individual’s cognitive and physical performances, mood, quality of life, social interaction and can lead to a decreased work productivity and increased injury risk too [6, 22, 23]. These consequences in response to sleep restriction or sleep disturbances are severe enough to research which is the best surface available to promote a night-time quality sleep. Previous studies reported that the mechanical characteristics of the mattress can play a key role for sleep quality. However, the existing data are still controversial [24]. Mattress firmness seems to have an effect since different studies showed that medium-firm mattress might reduce pain [25] and medium firmness bedding systems are correlated with higher sleep quality [26]. In line with this, two recent systematic reviews [24, 27] evaluating the effect of mattress design on sleep quality and pain concluded that medium-firm mattresses are beneficial for individual’s sleep and comfort and, in addition, these kinds of surfaces are typically perceived as more comfortable than soft bedding systems. Nevertheless, the spine alignment on different sleep surfaces has been little investigated in the past. To the best of our knowledge, this is the first study assessing the changes of the radiological alignment of the lumbar spine in supine position between two conditions: on a mattress *versus* a rigid surface. Further, we also evaluated possible correlations of lumbar angles between a standing and a supine condition (both in MAT and CON).

The main findings of this study were (i) L1**–**L5 and SS were greater in MAT than CON; (ii) L5**–**S1 was lower in MAT than CON; (iii) L1**–**S1 was greater in MAT than CON only for male subjects; iv) significant and positive correlations between PI and L1**–**L5, L1**–**S1, and SS were observed. Our initial hypotheses were only partially confirmed. The mattress used in the study was composed of a single layer of polyurethane and is typically considered a medium firm mattress. In the comparison between CON and MAT, significant differences were observed for three variables (*i.e.*, L1**–**L5, L5**–**S1, and SS) but not for the L1**–**S1 angle. Namely, L1**–**L5 and SS increased when participants were on the mattress, indicating that the mattress significantly influenced some of the lumbar angles, ultimately contributing to an increased sense of comfort. The underlying mechanisms explaining these differences are likely related to effect of mattress firmness on muscular stiffness and pressure distribution when lying supine [28]. Noteworthy is that we observed a different trend in superior and inferior lumbar angles: upper lordosis (*i.e.*, L1**–**L5) increased whereas lumbosacral lordosis (*i.e.*, L5**–**S1) decreased when subjects were laying down on the mattress compared to the control condition. Further, L1**–**L5 angle, but not L5**–**S1, positively correlated with PI in standing position for both MAT and CON, with no significant differences between the two conditions. This result is in line with previous studies showing that the proximal lumbar lordosis has a stronger correlation with PI than distal lumbar lordosis, both in standing and supine positions [29, 30].

For what concerns gender differences, male participants showed a significant increase in L1**–**L5, L1**–**S1, and SS angles in MAT whereas female subjects registered a significant increase only in SS. These results are only partially in line with previous studies showing that range of motion of spine segments during different motion tasks is significantly greater for females than for males [31]. Further, it has also been shown that, in static position, the mean values for lumbar lordosis and sacral slope are different between females and males, but a definitive consensus has not yet been found. Gelb et al. [32] assessed one-hundred healthy participants by a standing lateral radiograph of the entire spine and the authors observed that female subjects had a significant greater segmental lordosis at L2**–**L3, L3**–**L4, and L4**–**L5 compared to males. Legaye et al. [33] reported that men had greater L1**–**L5 values than women (61.4° ± 10.2° *versus* 58.1° ± 10.8°) while Bailey et al. [34] evaluated 200 healthy adults and found that lumbar angle was 7.3° greater in women than men in a standing position (60.3° ± 1.6° *versus* 53.0° ± 1.4°). Korovessis et al. [35] found that men had a higher sacral inclination than women (38° ± 10° *versus* 43° ± 12°). Conversely, the lordosis angle was not significantly different in a supine position (females, 49.4° ± 1.5°; males, 46.5° ± 1.7°; *p* = 0.208). On the contrary, our data suggest no significant differences between sex in L1**–**L5 angle (females, 46.0° ± 9.2°; males, 44.4° ± 8.4°), L1**–**S1 angle (females, 57.6° ± 7.4°; males, 55.3° ± 9.7°), SS (females, 38.3° ± 10.6°; males, 35.6° ± 6.4°), and PI (females, 47.0° ± 11.8°; males, 49.8° ± 7.3°).

One strength of this study is that both intra- and inter-rater reliability were tested for each outcome in MAT and CON. In detail, the intra-rater ICC was classified as excellent (> 0.9) for each variable, while the inter-rater ICC was classified as good (0.75–0.9) in seven variables and excellent in three variables (L1**–**S1 in CON, SS in CON, L1**–**S1 in EOS). Second, a validated assessment with MRI was used in the study so the high quality of the data makes the results reliable and repeatable. In addition, x-ray-based images were acquired with the EOS imaging system, which allows for low-dose biplanar images obtained in standing position. The full trunk, femoral heads, and the pelvis were included in the images with the advantage of having a non-conical projection that is typical of standard x-ray studies. PI, L1**–**L5, L1**–**S1 and SS indexes were therefore measured in a highly reproducible way [36]. PI is a position-independent parameter used to quantify spinopelvic sagittal balance [37], and has the characteristic of being an anatomical parameter that is independent of patient position and posture. In the present study, we observed, as expected, that PI had a significant and positive correlation with L1**–**L5, L1**–**S1, SS, but not with L5**–**S1, measured in both CON and MAT.

Some limitations need to be acknowledged. First, only one type of mattress was used in the study and the patients were evaluated only in the supine position; the results could change by using a mattress with different characteristics (material, density and/or structure) or with the patient laying in a prone position. Second, the sample size is small in the study (*n* = 20); however, the participants were recruited on the basis of specific inclusion and exclusion criteria, therefore the study sample was very homogenous, and the gender distribution was equal (10 males, 10 females). Additionally, we evaluated how the spinal alignment changed in the very short term, but we don't know how the column behaves may have changed after a full night of sleep. It is likely that the muscles relax with subsequent greater change in spinal angles. Further studies are warranted to evaluate this aspect.

In conclusion, this is the first study describing, with an accurate protocol and high-quality evaluations (*i.e.*, MRI), the changes of lumbar angles comparing rigid surface and mattress. We observed that the use of a mattress determines small but statistically significant changes in radiological angles describing the sagittal alignment of the lumbar spine when lying in the supine position. Our results do not have a direct clinical impact but they could represent the basis of future studies for the improvement of comfort and sleep quality. Authors also highlight the importance to design short- and long-term randomized controlled trials aiming to study spine alignment, sleep quality, and pain using different sleeping surfaces with various levels of firmness.

## Data Availability

The dataset of the study is available on the following URL: https://zenodo.org/record/5751798#.YqL3zKhBy5d.

## Abbreviations

BMI: Body mass index
CI: Confidence interval
CON: Control condition
ES: Effect sizes
ICC: Intraclass correlation coefficient
MAT: Mattress condition
MRI: Magnetic resonance imaging
PI: Pelvic incidence
SS: Sacral slope
